# Long-term course of anterior spinal cord herniation presenting with an upper motor neuron syndrome: case report illustrating diagnostic and therapeutic implications

**DOI:** 10.1186/s12883-020-01891-1

**Published:** 2020-08-29

**Authors:** Martin Regensburger, Johannes C. M. Schlachetzki, Jörg Klekamp, Arnd Doerfler, Jürgen Winkler

**Affiliations:** 1 Department of Molecular Neurology, University Hospital Erlangen, Schwabachanlage 6, 91054 Erlangen, Germany; 2 Center for Rare Diseases Erlangen (ZSEER), University Hospital Erlangen, Erlangen, Germany; 3 Department of Stem Cell Biology, University Hospital Erlangen, Erlangen, Germany; 4 Department of Neurosurgery, Christliches Krankenhaus, Quakenbrück, Germany; 5 Department of Neuroradiology, University Hospital Erlangen, Erlangen, Germany

**Keywords:** Spinal cord herniation, Evoked potentials, Spasticity, Case Report

## Abstract

**Background:**

Anterior spinal cord herniation (aSCH) is a rare cause of myelopathy which may present as pure motor syndrome and mimic other degenerative diseases of the spinal cord. In slowly progressive cases, diagnosis may be impeded by equivocal imaging results and mistaken for evolving upper motor neuron disease. As early imaging studies are lacking, we aimed to provide a detailed description of imaging and neurophysiology findings in a patient with aSCH, focusing on the early symptomatic stages.

**Case presentation:**

We here present the case of a 51-year old male patient with an episode of pain in the right trunk and a normal spinal MRI. After a symptom-free interval of 8 years, spasticity and paresis evolved in the right leg. There was subtle ventral displacement and posterior indentation of the thoracic spinal cord on MRI which, in retrospect, was missed as an early sign of aSCH. After another 3 years, symptoms spread to the left leg and a sensory deficit of the trunk became evident. Follow-up MRI now clearly showed an aSCH. Neurosurgical intervention consisted of remobilization of the herniated spinal cord and patch closure of the dura defect. Over the following years, motor and sensory symptoms partially improved.

**Conclusions:**

The history of this patient with aSCH illustrates the importance of careful longitudinal clinical follow-up with repeated imaging studies in progressive upper motor neuron syndromes. Specific attention should be paid to a history of truncal pain and to MRI findings of a ventrally displaced spinal cord. Neurosurgical intervention may halt the progression of herniation.

## Background

In early stages of slowly progressive spasticity of the legs, establishing a diagnosis may be challenging as symptoms are often limited to just one region. The diagnosis of motor neuron diseases like amyotrophic lateral sclerosis (ALS), hereditary spastic paraplegia and primary lateral sclerosis relies on the exclusion of spinal stenosis and inflammatory diseases of the central nervous system. This clinical scenario is reflected by the category “clinically possible ALS” according to the El Escorial criteria, defined by clinical evidence of upper and lower motor neuron dysfunction in one region only [1]. Patterns and time course of progression of motor neuron diseases show a considerable variation and cannot be sufficiently predicted to date [2]. Thus, while early diagnosis is an important prerequisite to initiate the correct type of treatment (e.g., riluzole, immunomodulation or surgery), slowly progressive upper motor neuron syndromes may remain unresolved during initial follow-up [3]. Moreover, clear-cut clinical signs or progression markers of upper motor neuron dysfunction are often lacking [4].

We herein report the case of a male patient with thoracic anterior spinal cord herniation (aSCH) who initially presented with monomelic spasticity and weakness leading to a suspected diagnosis of motor neuron disease. With a comprehensive demonstration of the long-term course of MRI and motor/ sensory evoked potentials, we provide a detailed description of potential early indications for this rare mimic of motor neuron disease.

## Case presentation

**Fig. 1:**
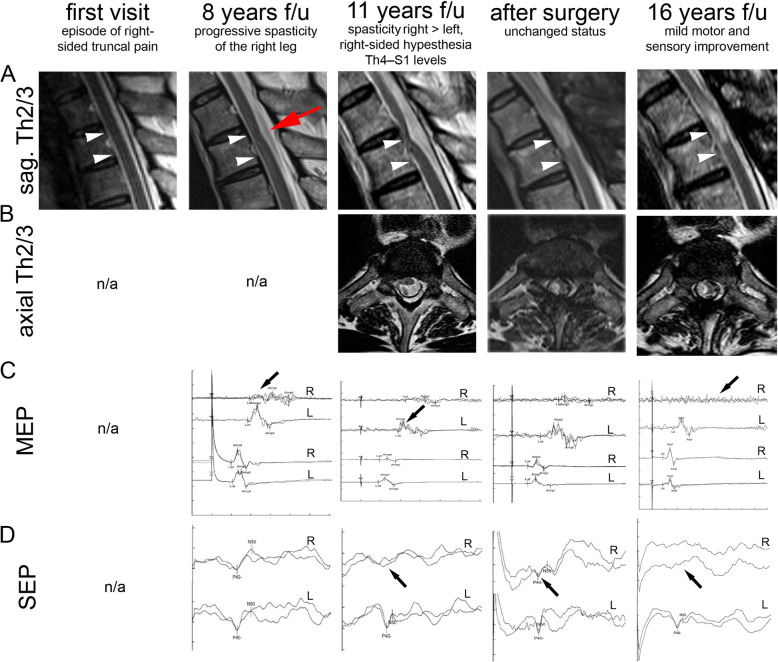
Longitudinal course of anterior spinal cord herniation. **a** Magnifications of the Th2/Th3 segment obtained in T2-weighted sagittal imaging at different visits. Arrowheads highlight evolving anterior spinal cord herniation and a postoperative myelopathy signal. The red arrow highlights the early subtle sign of ventral spinal cord misplacement and posterior indentation. **b** Corresponding axial images of Th2/3. **c** MEP including transcranial (lines 1–2) and lumbar stimulation (lines 3–4) to the right (*R*) and to the left (*L*) tibialis anterior muscle. Central motor conductance was pathological after 8 years follow-up (arrow; amplitude *R* 1.27 mV, *L* 2.88 mV). After 11 years, spasticity had progressed to the left leg, along with affection of the left leg central motor conductance (arrow; *R* 1.0 mV, *L* 2.47 mV). Six months after neurosurgery, there were no significant changes in MEP (*R* 0.68 mV, *L* 3.02 mV). Five years after neurosurgery, cortical MEP were absent to the right leg (arrow; *R* –, *L* 2.59 mV), despite unchanged MRI. **d** SEP-recordings during follow-up visits, obtained from stimulation of the right (*R*) and left (*L*) tibial nerve. At 8 years follow-up, SEP P40-latency was prolonged bilaterally (*R* 51.8 ms, *L* 52.0 ms). At presentation with Brown-Séquard syndrome involving right-sided hypesthesia, SEP from the right was absent (arrow; *R* –, *L* 50.8 ms). Six months after neurosurgery, SEP from the right gradually improved (arrow; *R* 51.2 ms, *L* 51.2 ms). Five years after neurosurgery, SEP from the right leg was absent again (arrow; *R* –, *L* 53.6 ms), despite unchanged MRI. *Scale*: C, 20 ms/ Div. and 2000 μV/ Div.; D, 20 ms/ Div. and 1.0 μV/ Div. *f/u* follow-up, *n/a* not available

A 51-year-old patient presented in 2004 with pain of the right side of the trunk. Neurological examination and spinal MRI (Fig. 1a) were normal. Symptoms resolved over a course of several weeks after physiotherapy and analgesics. Eight years later, in 2012, the patient developed progressive spastic paresis of the right leg, associated with a positive Babinski sign. Spinal MRI showed a lumbar disk protrusion bilaterally contacting S1 nerve roots, but was otherwise reported normal (Fig. 1a). Motor evoked potentials (MEP, Fig. 1c) indicated central motor conductance pathology to the right leg only. Somatosensory evoked potentials (SEP, Fig. 1d) were normal. Electromyography revealed fasciculations and chronic neurogenic changes in muscles innervated by nerve roots L4–S1 on the right side. Cerebrospinal fluid analysis was unremarkable. Accordingly, we diagnosed a degenerative upper motor neuron syndrome. Due to denervation patterns in EMG readings, differential diagnosis included early stages of ALS; furthermore, spastic paraplegia and primary lateral sclerosis were considered.

**Fig. 2:**
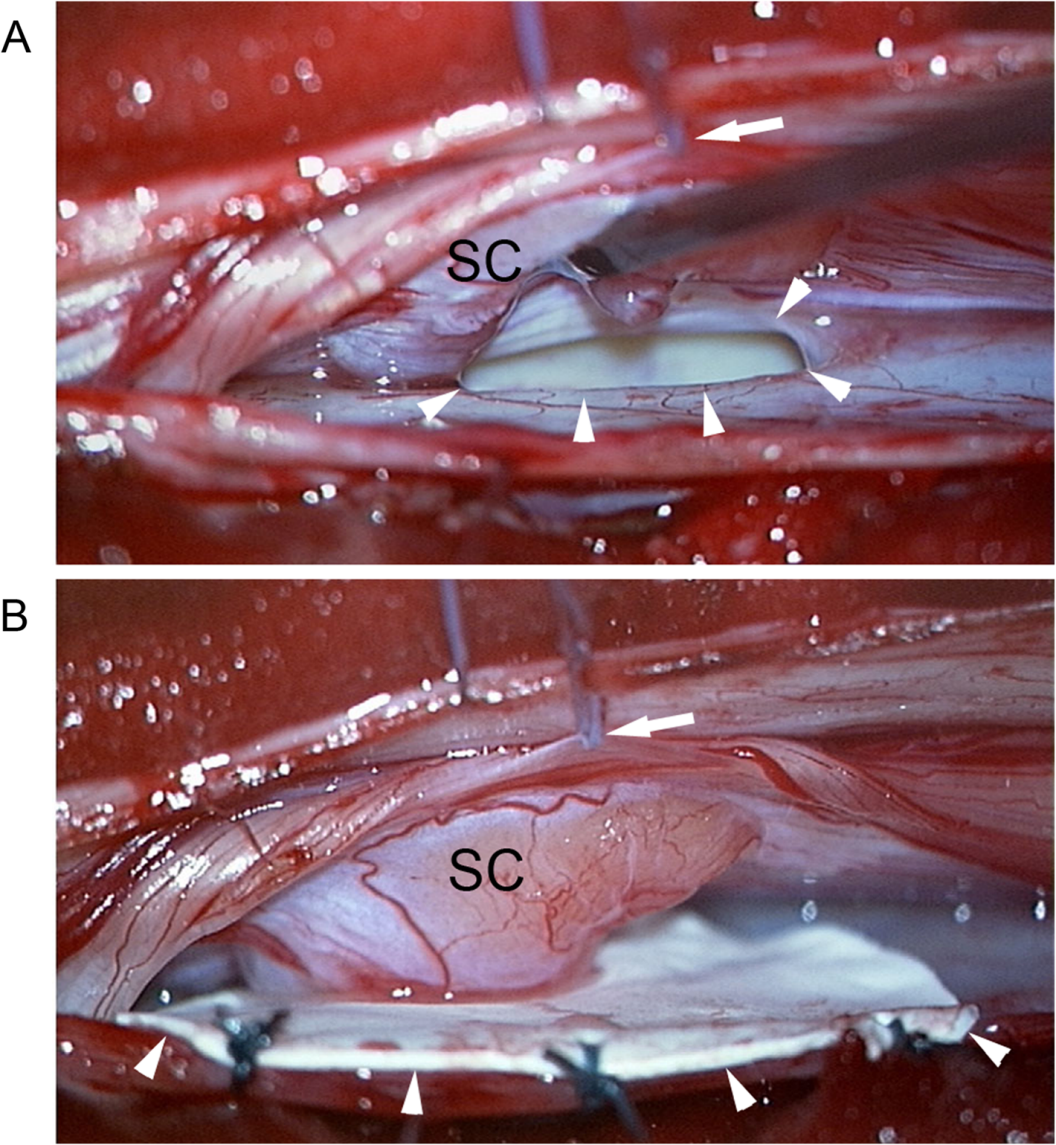
Intraoperative images. The intraoperative images demonstrate the defect in the anterior dural sac (arrowheads in **a**) after gently mobilizing the spinal cord with a suture applied to the dentate ligament (arrows in **a** and **b**) and closure of the defect with a dura patch (arrowheads in **b**). The herniated part of the spinal cord appears dysplastic (*SC* in B). *SC* spinal cord

Three years later, right-sided hypesthesia involving dermatomes Th4–S1 emerged subacutely, now matching with a Brown-Séquard syndrome. Follow-up spinal MRI showed an aSCH at Th2/3 (Fig. 1a-b). In retrospect, subtle signs of aSCH were already detectable in 2012: The spinal cord was ventrally displaced, appeared attached to the ventral dura and showed a posterior indentation at Th2/3 (Fig. 1a, red arrow). Due to the chronically progressive motor and sensory deficits, neurosurgery was performed. The neurosurgical treatment aimed at relocating the spinal cord into its normal intradural position with closure of the ventral dural defect to prevent reherniation. To gain sufficient access, the spinal cord was mobilized on the right side by applying sutures to the dentate ligaments (Fig. 2a). Part of the herniated cord could be lifted out of the defect easily, while the caudal part required sharp dissection before it could be mobilized. Finally, the defect was closed by muscle and fibrin glue. To prevent adhesions between cord and the closed dura defect, a goretex-patch was placed to cover the former defect (Fig. 2b). During surgery, SEP and MEP were monitored [5]. Postoperative MRI confirmed the corrected position of the spinal cord in the dural sac, and showed profound intramedullary signal changes consistent with a myelopathy at the level of former herniation (Fig. 1b). Six months after surgery, amplitudes and latencies of MEP were unchanged, whereas SEP was slightly improved (Fig. 1c, d). Clinically, symptoms improved partially over the course of 5 years after surgery. Spasticity of both legs was decreased (reduction of the modified Ashworth score of adductors from 2 to 1 on both sides) and the maximum walking distance was increased from 500 to 2000 m. The sensory deficit improved, but persisted in the right trunk (dermatomes Th2–L1). At the latest follow-up in 2020, the patient reported recurring stiffness and pain of both legs probably related to cessation of physical therapy due to COVID-19 pandemic restrictions. Interestingly, while MRI showed no signs of progression at this point, MEP correlated with the clinical increase in spasticity (Fig. 1c, d).

## Discussion and Conclusions

ASCH is a rare cause of myelopathy which may be missed on imaging during early stages of the disease [6]. Whereas Brown-Séquard syndrome is initially observed in the majority of aSCH cases, about 20% present with pure motor symptoms accompanied by spasticity and paresis of one or both legs [6]. In retrospect, specific signs merit attention as early indicators of a symptomatic spinal cause of lower limb spasticity: (i) Although the patient exhibited an isolated motor syndrome without sensory symptoms, the history of lateralized pain of the trunk should prompt the careful revision of MRI. (ii) Ventral displacement and posterior indentation of the spinal cord on the sagittal plane are indicative for aSCH and should trigger additional axial imaging (Fig. 1a, red arrow).

In the patient presented, coexisting multisegmental lumbar radiculopathy caused additional lower motor neuron pathology. Consequently, an early stage of motor neuron disease (ALS) could not be excluded at this point. SEP were prolonged bilaterally in this patient, but the finding of pathological SEP is not uncommon in ALS and did not preclude a diagnosis of ALS [7]. One patient with concomitant ALS and aSCH has been reported previously, but this single case may also have been coincidental and further follow-up of our patient clearly ruled out ALS [8].

So far, there is no widely accepted pathophysiological concept for these ventral dural defects. Degenerative changes, developmental defects, and spinal trauma may predispose to aSCH, but MRI studies prior to symptom onset are lacking [9]. Our imaging study reveals a gradual herniation of the spinal cord into a dural defect over a course of at least 3 years. The present course suggests that the increasing dural defect was related to degenerative changes. These include small bony spurs or calcified osteochondroses that may cause dural tears leading to larger defects and cord herniation [10]. This view is supported by the fact that the defects tend to be at the level of intervertebral spaces and that large calcified thoracic disc herniations tend to perforate the dura [11–13]. However, degenerative changes of the corresponding disc are not a regular feature in aSCH and were also not observed in the presented patient. It is also not known why aSCH almost exlusively affects the thoracic spine. Therefore, many authors consider these defects as a congenital disposition.

**Fig. 3:**
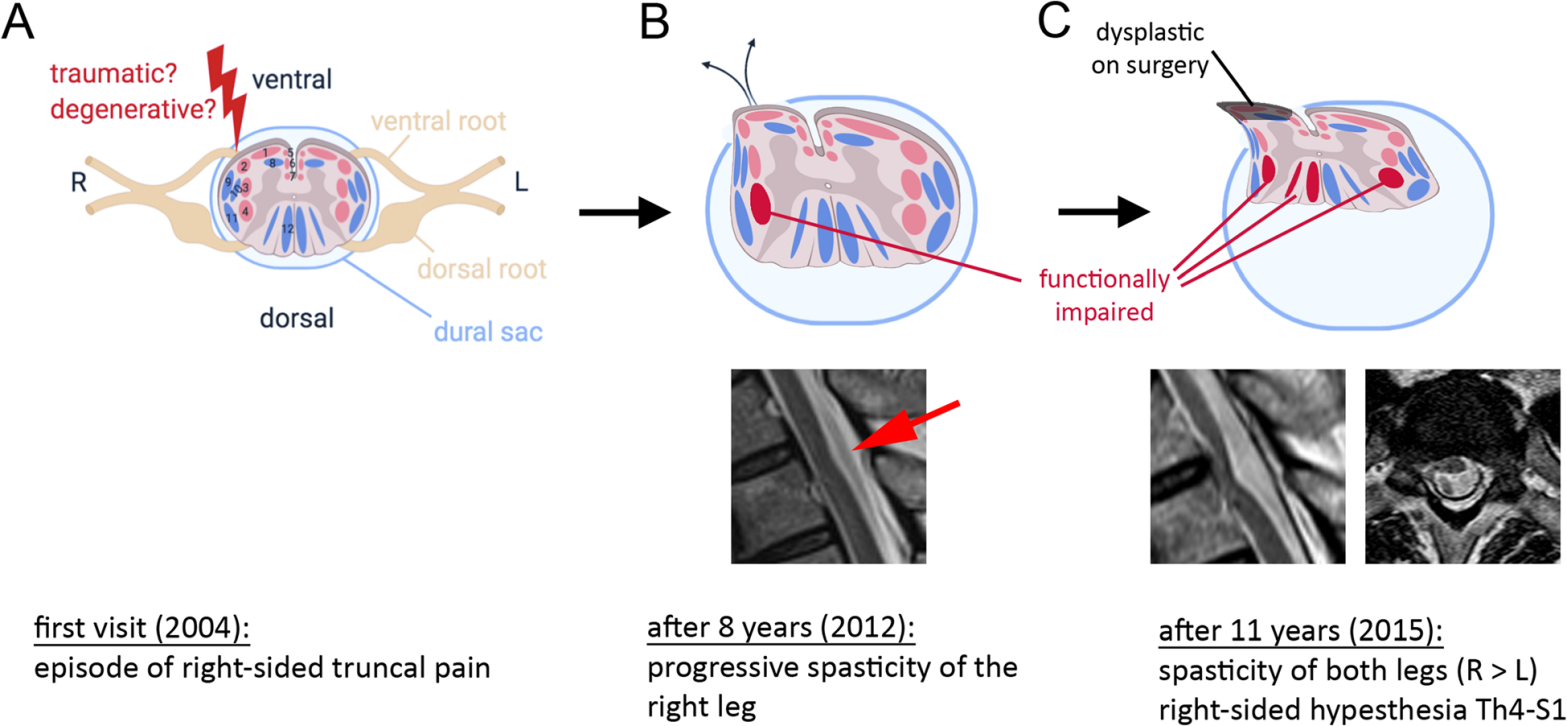
Schematic depiction of progressive anterior spinal cord herniation. **a** Schematic drawing of the central position of the thoracic spinal cord within the dura. The ventral dural defect may have been caused by congenital, traumatic and/ or degenerative changes. **b** The 8 years follow-up MRI showed ventral displacement of the spinal cord (red) as an early sign of aSCH without sensory symptoms. **c** When sensory symptoms evolved and surgery was performed, a substantial part of the anterior hemicord was herniated through the dural defect. *1* Vestibulospinal tract, *2* Olivospinal tract, *3* Rubrospinal tract, *4* Lateral corticospinal tract, *5* Tectospinal tract, *6* Anterior corticospinal tract, *7* Anterior reticulospinal tract, *8* Anterior spinothalamic tract, *9* Anterior spinocerebellar tract, *10* Lateral spinothalamic tract, *11* Posterior spinocerebellar tract, *12* Fasciulus gracilis

In case, symptoms progress or are associated with a functional impairment, neurosurgical intervention is indicated to halt worsening of the herniation in order to limit the functional deficit. In most cases, functional improvement was reported up to several years after neurosurgical intervention [6]. However, improvement depends on the affected fiber tracts (Fig. 3). Functional impairment is caused by different mechanisms which include pressure/ traction forces, hypoperfusion, secondary swelling, and tissue remodeling [14]. This explains the severe phenotype although only a part of the spinal cord is herniated. Segmental symptoms caused by anterior horn pathology at the Th2/3 level (e.g., intercostal weakness) were not observed, most likely due to the overlapping myotomic organization of the thoracic segments [15].

Postoperative segmental myelopathy, as seen in the presented patient, is commonly observed after spinal cord surgery. It is most likely caused by decompression or loss of traction forces acting on the myelon [6, 16]. As the myelopathy was stable over several years and there was no spinal cord atrophy, we did not suspect other causes of myelopathy (e.g., inflammatory, neurodegenerative) [17]. Levels of vitamin B12 and folic acid remained within normal limits during the postoperative course.

In summary, our patient illustrates the importance of careful longitudinal follow-up with repeated imaging studies in atypical clinical courses of suspected degenerative upper motor neuron syndromes.

## Data Availability

All data supporting our findings are presented within Fig. 1. Complete imaging results are upon file with MR and AD, and will be shared upon reasonable request.

## Abbreviations

ALS: Amyotrophic lateral sclerosis
aSCH: anterior spinal cord herniation
f/u: follow up
L: Left
MEP: Motor evoked potentials
n/a: not available
R: Right
SEP: Sensory evoked potentials
SC: Spinal cord
